# Disentangling the Potato Tuber Moth-Induced Early-Defense Response by Simulated Herbivory in Potato Plants

**DOI:** 10.3389/fpls.2022.902342

**Published:** 2022-05-26

**Authors:** Zhiyao Mao, Yang Ge, Yadong Zhang, Jian Zhong, Asim Munawar, Zengrong Zhu, Wenwu Zhou

**Affiliations:** ^1^State Key Laboratory of Rice Biology, Ministry of Agricultural and Rural Affairs Key Laboratory of Molecular Biology of Crop Pathogens and Insect Pests, Institute of Insect Sciences, Zhejiang University, Hangzhou, China; ^2^Hainan Institute, Zhejiang University, Sanya, China

**Keywords:** plant–insect interaction, defense phytohormone, simulated herbivory, insect oral secretion, insect-associated bacteria

## Abstract

Plants rely on the perception of a multitude of herbivory-associated cues (HACs) to activate their defense response to insect herbivores. These stimuli are mainly derived from three functional components, namely, mechanical damage, insect-associated microbe, and insect’s chemical cues. While simulated herbivory integrating these stimuli is widely exploited for complementing actual herbivory in clarifying the details of plant–herbivore interaction, breaking down these stimuli and identifying the mechanisms of plant responses associated with them have been less explored. In this study, the components of potato tuber moth (*Phthorimaea operculella*, PTM) herbivory were reorganized in a cumulative way and their impacts on the early defense responses of potato leaf were characterized. We found that simulated and actual herbivory of PTM triggered similar patterns of phytohormonal and transcriptomic responses in potato leaf. Moreover, the microbe in the PTM herbivory stimuli is associated with the regulation of the phytohormones jasmonic acid (JA) and abscisic acid (ABA) since reducing the microbe in HAC could reduce JA while increasing ABA. In addition, seven robust gene modules were identified to illustrate how potato plants respond to different PTM herbivory stimuli when herbivory components increased. Significantly, we found that mechanical damage mainly activated JA-mediated signaling; PTM-derived HACs contributed much more to potato early-defense response and induced signaling molecules such as multiple protein kinases; orally secreted bacteria stimuli could antagonize PTM-derived HACs and modulate plant defense, including repressing phenylpropanoid biosynthesis. Our study broadened the understanding of how potato plants integrate the responses to a multitude of stimuli upon PTM herbivory and evidenced that insect-associated microbes greatly modulated the plants response to insect herbivory.

## Introduction

Plants have evolved sophisticated strategies to tailor their defense responses to insect herbivores and optimize their fitness in nature. The specific recognition of insect attack requires the plant’s ability to perceive a multitude of stimuli derived from different functional components of insect herbivory (Schuman and Baldwin, 2016; Waterman et al., 2019). Upon herbivory, the mechanical damage breaks down plant cells and causes the releasing of many cell wall fragments and intracellular components, which were further recognized by specific receptors in plants (Choi et al., 2014; Merz et al., 2017). Meanwhile, insects release various herbivory-associated cues (HACs, Table 1) *via* their oral secretions (or regurgitant) to the wounds (Acevedo et al., 2015), and a variety of them have been identified and functionally characterized in different herbivores. Among them, the fatty acid–amino acid conjugates (FACs) (Alborn et al., 1997; Halitschke et al., 2001), the insect-derived peptides inceptins (Schmelz et al., 2006, 2007), and the fatty acid caeliferins (Alborn et al., 2007) are commonly defined as “elicitors” that activate plant defense response, whereas the glucose oxidases (GOXs) are usually found as “effectors” that inhibit plant defense (Lin et al., 2021).

**TABLE 1:** The abbreviations used in this chapter.

Abbreviation	Explanation
HAC	Herbivory-associated cue
PTM	Potato tuber moth
OSB	Orally secreted bacteria
FAC	Fatty acid-amino acid conjugate
GOX	Glucose oxidase
PAMP	Pathogen-associated molecular pattern
MAPK	Mitogen-activated protein kinase
ROS	Reactive oxygen species
JAZ	JASMONATE-ZIM DOMAIN
TF	Transcription factor
AAEE	Acetic acid ethyl ester
MRM	Multiple reaction monitoring
qRT-PCR	Quantitative real-time PCR
TPM	Transcript per million
PCA	Principal components analysis
DEG	Differentially expressed gene
GO	Gene Ontology

In addition to those insect- or plant-directly derived stimuli, a specific group of HACs from the herbivore-associated microorganisms can also modulate plant defense response. In the interaction between plant–microbe-vector insect, it was widely reported that pathogen-derived molecules, including pathogen-associated molecular patterns (PAMPs) and effectors, can intervene with herbivory-induced defense in plants (Kazan and Lyons, 2014; Toruno et al., 2016). Recently, many evidence suggested that some microorganisms within the herbivore oral secretion are indispensable for insects to modulate host defense response. For instances, *Helicoverpa zea* gut-associated bacteria *Enterobacter ludwigii* can trigger salivary elicitors and indirectly induce tomato defense (Wang et al., 2017). The Colorado potato beetle (*Leptinotarsa decemlineata*) exploits orally secreted bacteria (OSB) to suppress tomato defense (Chung et al., 2013). However, the mechanism of how these microbes assist insects in modulating plant defense response remains to be clarified.

After the perception of herbivore stimuli, plants rapidly initiate the defense signaling cascades [mitogen-activated protein kinase (MAPK), Ca^2+^, reactive oxygen species (ROS), etc.] and activate the phytohormones and regulatory gene networks to reconfigure their metabolites (Altmann et al., 2020). Although jasmonic acid (JA) and salicylic acid (SA) are the main hormones in the defense against herbivores, other hormones such as abscisic acid (ABA) are also involved (Verma et al., 2016). JA derivatives (JA-Ile, etc.) trigger the degradation of JASMONATE-ZIM DOMAIN (JAZ) and the subsequent derepression of multiple transcription factors (TFs), which further initiates the expression of many herbivore defense genes (Erb and Reymond, 2019). Moreover, the interaction of phytohormones helps refine the effect of these active chemicals in plant–herbivore interaction. For instance, SA signaling usually antagonize JA signaling and turn down its impact (Petersen et al., 2000; Spoel et al., 2003), whereas ABA works in action with JA in many plants (Thaler and Bostock, 2004; Bodenhausen and Reymond, 2007).

**FIGURE 1:**
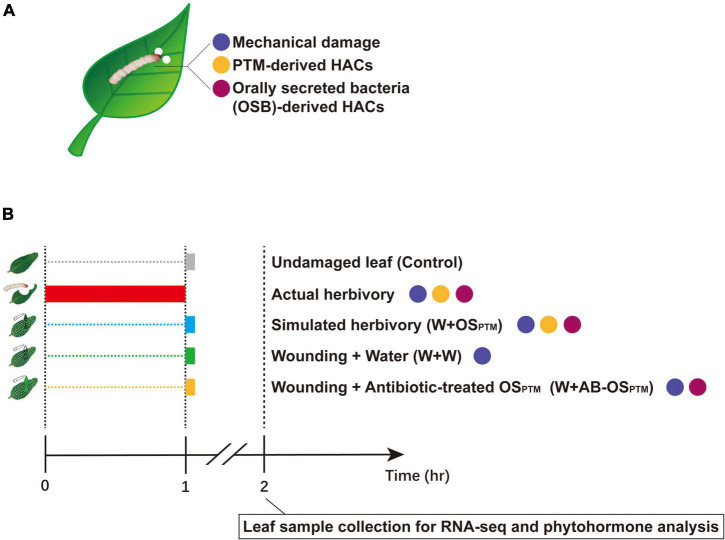
Breaking down PTM herbivory and reorganizing its components. **(A)** PTM herbivory contains three types of stimuli: mechanical damage, PTM-derived HACs, and OSB-derived HACs. The differently colored dots refer to different types of stimuli. **(B)** The illustration of experiment setup: actual herbivory, W + OS_PTM_, W + W, and W + AB-OS_PTM_ combine different types of stimuli as shown; in the actual herbivory group, potatoes were intensively treated with PTMs for 1 h and other treatment groups were simultaneously treated at the end of the actual herbivory treatment. All samples were collected at 1 h after simulated herbivory treatments and taken to RNA-seq and phytohormone test.

The potato tuber moth (PTM), *Phthorimaea operculella* Zeller, is one of the most damaging pests of potatoes worldwide. PTM and its main host potatoes are both native to South America, and their long-term interaction likely affected the evolution of both species (Chandel et al., 2020). On one hand, PTM has well adapted to solanines and other defense metabolites in potato plants; on the other hand, some potato wild species and cultivars have specific defense machinery against this herbivore (Musmeci et al., 1997; Horgan et al., 2007; Mweetwa et al., 2012; Zhang et al., 2021). However, it remains unclear how potato plants sense the PTM herbivory to tailor their defense against this specialist herbivore, and how potato plants integrate the stimuli coming from different functional components of PTM herbivory. In this study, the simulated herbivory of PTM is exploited for complementing its actual herbivory in clarifying the details of the early-defense response of potato plants to PTM damage. We further reorganized the functional components of PTM herbivory stimuli (Figure 1A) in a cumulative way (Figure 1B), identified the gene modules of potato early-defense responses associated with different herbivory components, and revealed how potato plants integrated early response to PTM herbivory *via* transcriptomic and phytohormone analysis.

## Materials and Methods

### Plants

*Solanum tuberosum* group Tuberosum RH89-039-16 (RH; The Potato Genome Sequencing Consortium, 2011) seedlings were obtained *via* tissue culture following the procedure described by Rani et al. (2012) at 20 ± 1°C, 16 h light (100 μmol m^–2^ s^–1^; LED T5 21W). Three-week-old seedlings were transplanted into 1 L pots filled with peat moss potting soil (Shenzhen Shenglvyuan Horticulture Co., Ltd., China) and grew in greenhouse (22 ± 1°C, 16 h of light; 16 ± 1°C, 8 h dark) with a light intensity of 600–800 μmol m^–2^ s^–1^ (600W, Lucagrow, Hungary). Plants were watered every 4 days to keep soil humidity within 20–30%.

### Plant Treatment and Sample Preparation

*Phthorimaea operculella* herbivory stimuli are disassembled into three types of stimuli (Figure 1A) encompassing different herbivory components. PTM oral secretion (OS_PTM_) was collected from third-instar PTM larvae (the larvae were starved for 6 h and fed with RH leaf for 12 h) and then centrifuged at 5,000 rpm (4°C) for 5 min to remove pellet; OS was then 1:5 diluted with sterile ultrapure water before using. For antibiotic-treated PTM oral secretion (AB-OS_PTM_), potato leaves for feeding PTM larvae were treated with the antibiotic cocktail [per 50 ml of antibiotic cocktail contains 0.1 g neomycin sulfate (Macklin), 0.5 g aureomycin (Macklin), and 0.03 g streptomycin (Macklin)] as Chung et al. (2013) every 24 h for 48 h; AB-OS_PTM_ was then collected and centrifuged before using. To confirm that antibiotic cocktail does not affect phytohormonal responses, we treated potato leaves with a pattern wheel; and wounds were immediately treated with ultrapure water (W + W), antibiotic cocktail 1:5 diluent (W + AB diluent), and antibiotic cocktail (W + AB); as shown in Supplementary Figure 7, antibiotic cocktail did not affect phytohormonal responses of potato leaf. The presence of microbe in OS_PTM_ and AB-OS_PTM_ was checked by culturing them on 2× YT plate overnight, after which the colony numbers were counted (Supplementary Figure 1).

The second fully expanded young leaf of 3-week-old RH plant (post potting) was treated with different types of stimuli (Figure 1B). A total of 30 third-instar PTM larvae were clip-caged on potato leaf tissue and allowed to intensively feed for 1 h (actual herbivory). A pattern wheel was rolled with around 0.5 cm interval on leaf surface and the wounds were immediately treated with sterile ultrapure water (W + W) or OS_PTM_ (W + OS_PTM_) or AB-OS_PTM_ (W + AB-OS_PTM_). Each replicate of a treatment came from an independent potato plant. The leaf samples were collected and flash-frozen at 1 h after treatment; the samples were then stored at −80°C till usage.

### Phytohormone Measurement

The phytohormone extraction, measurement, and analysis were performed as described by previous studies (Kang et al., 2006; Wang et al., 2007; Luo et al., 2016). Briefly, samples were ground in liquid N_2_ into powder; 0.5 ml 80% methanol and 1 μl internal standard (10 ng/μl, D_4_SA, D_6_JA, D_6_ABA, D_6_JA-Ile) were added to 100-mg sample powder. The samples were vortexed and kept at 4°C overnight. The samples were dried with nitrogen gas, after which 200 μl acetic acid ethyl ester (AAEE) was added and vortexed for 5 min; the AAEE layer was transferred into a new 1.5 ml EP tube. The AAEE solution was dried with nitrogen gas and samples were then dissolved again with 100 μl 50% methanol. The samples were vortexed for 5 min and centrifuged at 10,000 rpm for 5 min; the samples were finally added into HPLC tubes for HPLC-MS/MS analysis.

Phytohormones were analyzed with Waters Xevo TQ-XS multiple reaction monitoring (MRM) mode. A C18 reversed-phase HPLC column was used for chromatography and following binary gradient was used, namely, 0–1.5 min, isocratic 60% A (deionized water, 0.1% formic acid), 40% B (methanol), 0.3 ml/min flow rate; 1.5–8 min, gradient phase to 100% B, 0.3 ml/min flow rate. Negative ion mode was used for MRM and the following setting was used [precursor (*m/z*)/product (*m/z*)/collision energy (eV)]: 137/93/10 for SA, 209.1/59.1/10 for JA, 263.1/153/10 for ABA, 322/130/20 for JA-Ile, 141/97/10 for D_2_SA, 215/59/10 for D_6_JA, 269.3/159/10 for D_6_ABA, and 328/130/20 for D_6_JA-Ile. Other settings followed default settings.

### RNA Extraction and RNA-seq

Samples were ground in liquid N_2_ into powder; 25–50 mg leaf powder was pooled for RNA extraction. We extracted total RNA from sample powder pools with Easy-Go Plant RNA Kit (TRIzol) according to the instruction of the manufacturer. The concentration and quality of RNA were tested with Thermo Fisher Scientific NanoDrop 2000 (A260/A280 = 1.8–2.1, A260/A230 = 1.8–2.0); high-quality RNA was sent to RNA-seq and used for quantitative real-time PCR (qRT-PCR) validation. For qRT-PCR analysis, the elongation factor gene *EF3d* were used as an internal control, the primers for qPCR are shown in Supplementary Table 1, and three biological replicates were used for each treatment.

The high-quality RNA samples were sequenced with Hiseq-PE150 platform, and 3–5 biological replicates were used. The raw data were converted to FASTQ format and cleaned. The cleaned FASTQ RNA data were aligned to potato genome with HISAT2; the resulting SAM data were converted to sorted BAM data with SAMtools. All RNA-seq reads data were deposited in European Nucleotide Archive (Accession: PRJEB51839). StringTie was used to count reads for each gene at last; the read count matrix was converted to transcript per million (TPM) expression matrix. The qRT-PCR result for selected genes consisted with the RNA-seq result (Supplementary Figure 5), indicating the high-quality RNA-seq.

Principal component analysis (PCA) for gene expression matrix was conducted with princomp() in R; the genes of low expression (TPM < 1) were filtered; and the expression matrix was scaled and centered before analysis. To quantify the inductivity for one treatment, we utilized relative distance plasticity index (RDPI) to calculate all possible pairings between control group and treatment group and the RDPIs represented the inductivity of the treatment (Valladares et al., 2006).

To get differentially expressed genes (DEGs), we first made hierarchical clustering (method = “average”) for all samples based on Euclidean distance of all genes and removed the outliers of each treatment. The gene expression of W + W, W + AB-OS_PTM_, W + OS_PTM_, and actual herbivory was analyzed using the R software “DESeq2” from Bioconductor (Gentleman et al., 2004; Love et al., 2014). The gene whose mean read count was less than 10 was removed. Genes were considered DEGs if their |log_2_FoldChange| > 1.5 and *p*-adjusted < 0.05.

### Gene Module Recognition

To recognize robust gene modules-associated different functional components and reveal how potato plants integrate response to PTM herbivory, three cumulative DEG analyses were conducted for the following pairs, namely, control and W + W, W + W and W + AB-OS_PTM_, W + AB-OS_PTM_, and W + OS_PTM_; the genes were then classified into 27 patterns by the results of three cumulative DEG analyses. Before analysis, the gene whose mean read count is less than 10 was removed. Genes were considered DEGs if their |log_2_FoldChange| > 1.5 and *p*-adjusted < 0.05.

### Function Enrichment Analysis

Gene Ontology (GO) enrichment was conducted with R software package “clusterProfiler” according to the instruction of the software (Yu et al., 2012). We used Jaccard’s coefficient to quantify the similarity between the GOs:


(1) $$J\,\left(A,B\right)=\frac{\left|A\cap B\right|}{\left|A\cup B\right|}.$$

where *A* is the potato gene set of GO term A, and *B* is the potato gene set of GO term B. Two GOs were considered connected if the *J*(*A*,*B*) > 0.2.

Functional network was built with top 15 significant GOs of each gene module. Intramodular connectivity of each gene module was quantified with cluster coefficient (C):


(2) $$C=\frac{1}{N}\,\sum_{N}^{1}C_{i},$$

(3) $$C_{i}=\frac{2\,e_{i}}{k_{i}\,\left(k_{i}-1\right)}.$$

where *C*_*i*_ is the cluster coefficient of a node in gene module; *e*_*i*_ is the number of closed triangles that the node and its neighbors contain; and *k*_*i*_ is the degree of the node. Extramodular connectivity was quantified with the sum of degree of the gene module. Gene modules were functionally clustered based on topological overlap (Zhang and Horvath, 2005).

### Statistics

The data of each treatment group were tested with Shapiro–Wilk test and Bartlet test to verify normality and homogeneity of variance; the data that did not pass two tests were transformed. ANOVA in the study was conducted with aov() in R; the multiple comparison was conducted with Tukey’s honestly significant difference method.

## Results

### Potato Plant Showed Different Phytohormonal Responses to Different Types of Potato Tuber Moth Herbivory-Associated Cues

**FIGURE 2:**
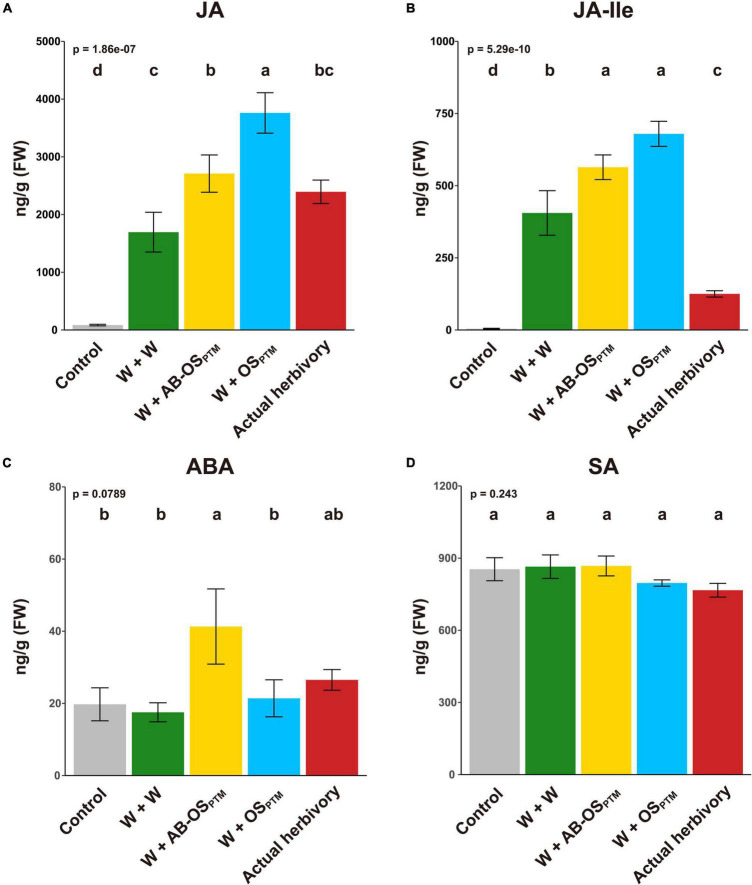
Phytohormone levels after the induction of different herbivory stimuli. The levels of four defense phytohormones **(A)** JA, **(B)** JA-Ile, **(C)** ABA, and **(D)** SA were measured in leaves at 1 h after all treatments (mean ± SE, *n* = 5). The *p*-value of ANOVA is displayed in the upper-left corner; the result of multiple comparison (Tukey’s HSD) is shown at the top of bars.

To understand how potato plants respond to different types of HACs from PTM (Figure 1), we first studied the phytohormone changes in leaves elicited by these HACs. The PTM actual herbivory and simulated herbivory (W + OS_PTM_) both significantly induced the accumulation of JA and JA-Ile (Figures 2A,B) but not ABA (Figure 2C) and SA (Figure 2D) in potato leaves. It is reasonable to see that PTM simulated herbivory (W + OS_PTM_) induced higher level of jasmonates than that of actual herbivory (Figures 2A,B), which might be caused by the different signal intensity between these two treatments. Moreover, mechanical damage (W + W) alone could induce the high accumulation of JA and JA-Ile (Figures 2A,B) but not ABA (Figure 2C) and SA (Figure 2D), suggesting an important role of this stimuli in triggering herbivore defense. Furthermore, introducing chemical cues from HACs (W + AB-OS_PTM_ and W + OS_PTM_) significantly enhanced the level of these two jasmonates in comparison to mechanical damage, and the W + OS_PTM_ treatment also induced their higher level than that of W + AB-OS_PTM_ (Figures 2A,B), indicating an additive effect of plant response to these three stimuli in terms of JA signaling. Interestingly, ABA is specifically induced by W + AB-OS_PTM_ treatment (Figure 2C), indicating the microbe in OS_PTM_ could suppress the accumulation of this phytohormone in potato plants.

### Potato Plants Showed Different Transcriptomic Responses to Different Types of Potato Tuber Moth Herbivory-Associated Cues

**FIGURE 3:**
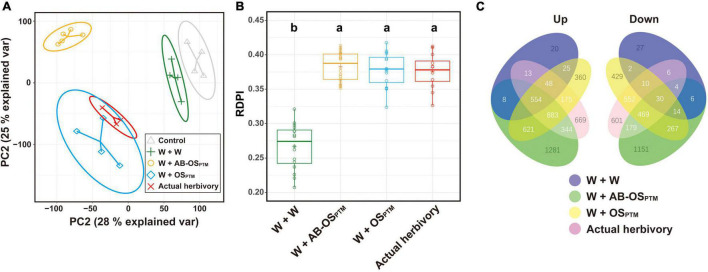
The comparison of transcriptomic profile for all treatment groups. **(A)** PCA for gene expression matrix; 28% variance is explained in PC1 and 25% variance is explained in PC2; ellipse refers to the 95% confidence interval of each treatment group. **(B)** The boxplot of RDPI index for four herbivory treatment groups; data point is mapped to circle. **(C)** DEG overlap and difference for all treatment groups are exhibited as a Venn plot; the number of genes of each region is displayed on the corresponding region.

Comparative transcriptomic analysis was further used to study the plant defense response variation in leaves elicited by different HACs. The transcriptomes of simulated herbivory (W + OS_PTM_) were highly overlapped with that of actual herbivory in the PCA, indicating a similar gene expression profile between these two treatments (Figure 3A). Moreover, the PCA can also differentiate the leaf transcriptomes treated by the three different herbivory stimuli W + W, W + AB-OS_PTM_, and W + OS_PTM_ (Figure 3A), suggesting the significant differences in expression profiling among these treatments. Interestingly, the W + AB-OS_PTM_ treatment is distantly away from the other treatments according to the PCA result (Figure 3A), which was also supported by the Venn plot of DEGs of these treatments (Figure 3C), indicating the specificity of transcriptomic response caused by the HACs in this type of stimuli. In addition, the inducibility of the transcriptomes treated by the three stimuli W + AB-OS_PTM_, W + OS_PTM_, and the actual herbivory was similar, which was significantly higher than that of the mechanical wounding treatment (Figure 3B), indicating that the chemical cues from HACs could greatly enhance the transcriptomic responses in comparison to the mechanical damage alone.

### Potato Tuber Moth Actual and Simulated Herbivory-Induced Similar Changes in Functional Gene Groups in Potato Plants

**FIGURE 4:**
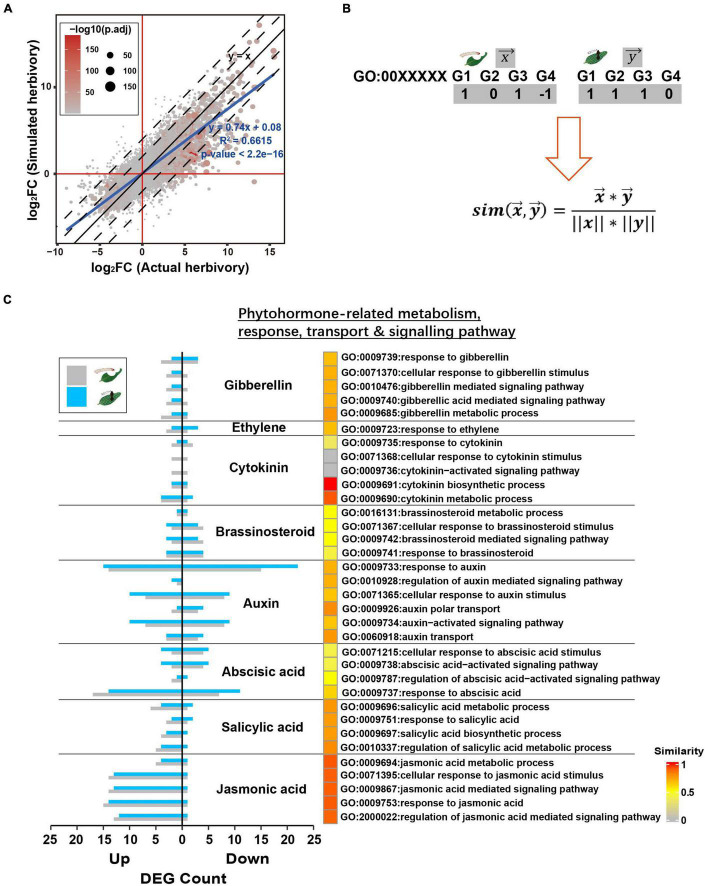
Simulated herbivory and actual herbivory induced similar early transcriptomic response of potato plants. **(A)** Log2(fold change) of all genes of simulated herbivory group and actual herbivory group was compared in a dot plot. If simulated herbivory and actual herbivory induced the same responses, all genes should fall in *y* = *x*. **(B)** The overview to quantify inductive similarity of two treatments in a GO; treatment *X* and *Y* induced DEGs in a GO (1, upregulated DEG; –1, downregulated DEG; 0, non-significant gene); the DEG vector *x* and *y* were plugged into equation to calculate similarity. **(C)** The inductive similarity between actual herbivory and simulated herbivory in all phytohormone-related GOs. The DEG number of simulated herbivory and actual herbivory in each GO is exhibited as bar plot. The similarity was mapped to heatmap color from gray to red.

To further assess the similarity between PTM actual herbivory and its simulated herbivory, we specifically compared the transcriptomes induced by these two stimuli. At a global level, the fold change (related to the undamaged control) of all genes in actual herbivory and simulated herbivory fitted the linear curve *y* = 0.74*x* + 0.08 (*R*^2^ = 0.6615, *p* < 2.2e−16), indicating the overall trends of transcriptomic responses were highly similar between these two treatments, whereas a less difference in response level was observed (Figure 4A).

Upon herbivory, phytohormone-centered early signal network further activates reconfiguration of primary metabolism and secondary metabolism. Thus, we selected key GOs related to specific defense responses and quantified similarity between these two treatments *via* cosine similarity of these GOs (Figure 4B). PTM herbivory and its simulated herbivory induced similar changes in phytohormone-related GOs, especially in JA and SA (Figure 4C). PTM herbivory and its simulated herbivory also both induced strong transcriptional-level changes in auxin-related functional genes, suggesting their common effect on potato growth (Figure 4C). In addition to phytohormone-related functions, PTM herbivory and its simulated herbivory commonly induced similar responses in other functions like photosynthesis, primary metabolism, and secondary metabolism (Supplementary Figure 2). In summary, these results further indicated that PTM herbivory and its simulated herbivory induced similar changes in the defense response of potato plants.

### Recognizing Herbivory-Associated Cue Functional Component-Associated Gene Modules Within Plant Defense Response Genes

**FIGURE 5:**
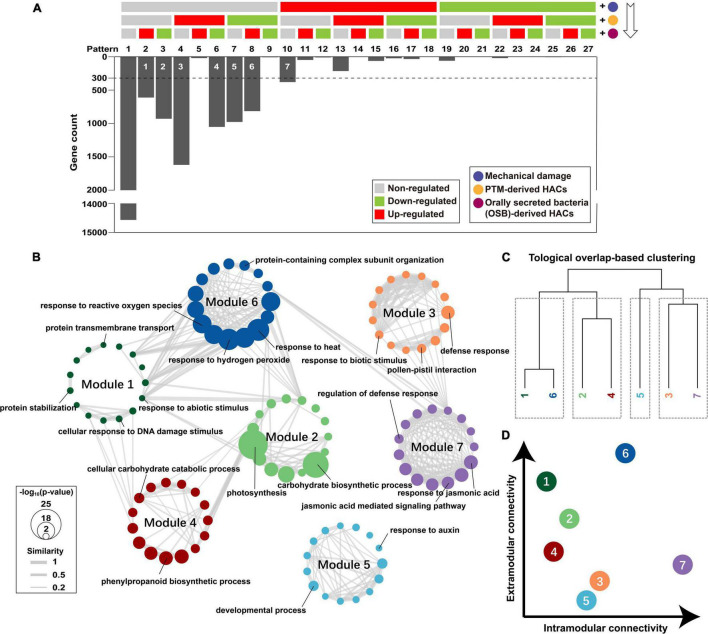
Dissecting how potato plants integrate responses to stimuli of different PTM herbivory components. **(A)** Seven robust gene modules associated with different components were identified (white number on the bars). The regulated genes were identified *via* DEG analysis every time component was added; and 300 was set as a threshold of minimal module size. **(B)** Top 15 overrepresented biological process GOs of each gene module were integrated as a functional network. Node refers to the GO term, and the edge refers to that two GOs were functionally connected; *p*-value of overrepresentation analysis is mapped to node size; Jaccard similarity between two GO terms is mapped to edge width; gene module is mapped to node color. **(C)** Gene modules were clustered based on topological overlap; it cut gene modules into four clusters. **(D)** The intramodular connectivity and extramodular connectivity of gene modules are displayed as a dot plot.

To reveal how potato plants integrate early responses for a multitude of stimuli upon PTM herbivory, we reorganized the functional components of PTM herbivory in a cumulative pattern (W + W, W + AB-OS_PTM_, and W + OS_PTM_) and demonstrated how genes were transcriptionally regulated when a functional component was added (Figure 5A). DEG analysis [| log_2_(FC) > 1.5|; *p*-adjusted < 0.05] was conducted every time a functional component was added to select robust responsive genes, which were further assigned into 27 theoretical gene expression patterns after three-time component addition. Practically, we found that 67.86% of the genes (14,566 of 21,466, pattern 1) in potato leaf did not show significant change in transcript accumulation levels upon the treatment of the three components (Figure 5A). The genes showed significant transcript response were highly biased in the distribution among the 26 patterns, in which more than 92.49% of responsive genes belongs to 7 patterns.

We then identified seven robust gene modules representing different gene transcript responses after the stimulation of three functional components (Figure 5A). Only module 7 (379 genes) contained genes that were specifically induced by mechanical damage. Module 3 (1,620 genes) and 5 (977 genes) contained genes that were specifically induced and suppressed by PTM-derived HACs, respectively. Modules 1 (611 genes) and 2 (929 genes) contained genes more specifically induced and suppressed by OSB-derived HACs, respectively. Modules 4 (1,051 genes) and 6 (815 genes) contained genes that responded to both PTM-derived HACs and OSB-derived HACs, whereas their expression patterns were opposite to each other. GO enrichment was further conducted for these gene modules, and the top 15 significant GO terms of each module were selected to build GO networks for biological process (Figure 5B) and molecular function (Supplementary Figure 4A). Then, these gene modules were clustered based on topological overlap (Zhang and Horvath, 2005; Figure 5C and Supplementary Figure 4B). In biological process network, modules 1 and 6, modules 2 and 4, and modules 3 and 7 were functionally linked, respectively, whereas module 5 was barely connected with other modules (Figure 5B). Modules 6 and 7 had high intramodular connectivity, whereas modules 1 and 6 had high extramodular connectivity (Figure 5D). In molecular function network, most of the gene modules had low intramodular connectivity, and modules 6 and 7 had relatively high intramodular connectivity (Supplementary Figure 4B).

Within each module, module 7 was enriched for GO terms with “response to jasmonic acid,” “jasmonic acid mediated signaling pathway,” “regulation of defense response,” etc. (Figure 5B and Supplementary Datasheet 2), indicating the mechanical damage alone could activate many JA-mediated defense responses in potato plants. Modules 3 and 5 were enriched for “response to biotic stimulus,” “defense response,” “developmental process,” “response to auxin,” etc. (Figure 5B and Supplementary Datasheet 2), suggesting that PTM-derived HACs not only regulate the plant defense response, but also affect plant development and growth. Modules 1 and 2 were enriched for “response to abiotic stimulus,” “protein stabilization,” “photosynthesis,” “carbohydrate biosynthetic process,” etc. (Figure 5B and Supplementary Datasheet 2), indicating that OSB-derived HACs could regulate various plant responses, including the primary metabolism. Modules 4 and 6 were enriched for “response to reactive oxygen species,” “response to hydrogen peroxide” “cellular carbohydrate catabolic process,” “phenylpropanoid biosynthetic process,” etc. (Figure 5B and Supplementary Datasheet 2), suggesting that PTM-derived HACs and OSB-derived HACs both activate the ROS signaling and the secondary metabolism in potato plants.

## Discussion

Simulated herbivory has long been a practical method to simplify, standardize, and synchronize insect herbivory treatment in the plant–insect interaction studies. In this study, we systematically studied the potato plant response to the herbivory of the potato tuber moth *via* breaking down simulated herbivory stimuli and reorganizing its components in a cumulative way.

Potato plants stressed by simulated herbivory and actual herbivory showed similar phytohormonal and transcriptomic response patterns, whereas their signal intensity may vary. The transcriptomic response induced by actual herbivory was slightly stronger than that induced by PTM simulated herbivory, especially in the way of gene fold change levels (Figure 2C); while JA induction by simulated herbivory was stronger than that of actual herbivory did (Figures 3A,B). These results could be explained by two possible reasons. First, simulated herbivory lacks characteristics that are present in actual herbivory. For example, the mechanical vibration caused by insect herbivore chewing can be sensed by *Arabidopsis thaliana* (Appel and Cocroft, 2014; Kollasch et al., 2020), which is difficult to mimic in simulated herbivory. Second, actual herbivory is a continuous and cumulative stress, whereas simulated herbivory is a short and intense stress, thus the progress of plant response induced by actual herbivory treatment is faster than that of simulated herbivory, whereas we tried to avoid this by treating potato leaves with 30 third-instar PTM larvae for 1 h to cause intense and short herbivory. In any case, simulated herbivory could still be a useful strategy to complement the shortages of actual herbivory in practice.

The mechanism of how host plants sense mechanical damage and insect-derived HACs has been broadly revealed in many plants (Pieterse et al., 2012; Christensen et al., 2013; Schweizer et al., 2013; Song et al., 2014). Recent studies proved that insect-associated microbes, including bacteria, also played important roles in the modulation of host plant defense. This modulation came in at least two possible ways, namely, (1) microbes were secreted into wounds and directly modulated host plant defense that has been widely reported (Chung et al., 2013; Acevedo et al., 2017) and (2) microbes indirectly modulated host plant defense *via* changing insect-derived HACs (Wang et al., 2017). In this study, we broadened the understanding of how OSB modulate host plant defense.

With PTM potato model, we disentangled the early response of potato to PTM herbivory and demonstrated that OSB played important regulatory roles within this response. In *A. thaliana*, JA and ABA mainly exhibited synergistic effects on resistance to insect herbivory (Erb et al., 2012). In potato, we found that OSB-derived HACs strengthened JA induction (Figures 3A,B), whereas ABA induction was inhibited by OSB-derived HACs (Figure 3C), suggesting that OSB-derived HACs may inhibited ABA induction in a JA-independent way; meanwhile, the expression of biosynthetic genes of JA (Supplementary Figure 3A) and ABA (Supplementary Figure 3B) also supported the observation. Surprisingly, all treatments did not affect SA content, which suggested that OSB modulated potato resistance barely *via* JA–SA antagonism (Robert-Seilaniantz et al., 2011; Thaler et al., 2012).

Phenylpropanoid metabolism was reported to generate an enormous array of secondary metabolites such as flavonoids and benzoic acid (Vogt, 2010). In this study, gene module 2, which covered many secondary metabolism gene ontologies, especially the phenylpropanoid biosynthesis, was repressed by OSB-derived HACs. Moreover, phenylalanine ammonia lyase (*PAL*), as the first enzyme of phenylpropanoid metabolism, was also repressed by OSB-derived HACs. Thus, it is likely that OSB-derived HACs could repress phenylpropanoid metabolism *via* reducing the activities of key enzymes. It would be interesting to exploit how OSB modulate potato defense to PTM herbivory *via* the phenylpropanoids.

Recognizing gene modules among treatments or genetically different lines helps to identify the correlative genes that might be regulated in a similar pattern and provide clues to the identification of hub genes (Zhou et al., 2016; Bjornson et al., 2021). In this study, the early transcriptomic response of potato plants to a multitude of stimuli upon PTM herbivory was mainly grouped into seven robust gene modules. These modules could represent seven main patterns of how potato plants integrate responses to herbivory components, whereas their sizes reflect the regulative breadth of associated component. Moreover, intramodular or extramodular connectivity reflects the functional relevance within or between modules. Mechanical damage, as the most basic component of insect herbivory, induced potato early response (module 7), which was largely interconnected and mainly related to JA-mediated pathway, indicating that JA is a master regulator of plant response to wounding (Farmer and Dubugnon, 2009; Koo and Howe, 2009; Erb et al., 2012). PTM-derived HACs induced broad early response (modules 3–6) as previously reported (Schmelz et al., 2009; Acevedo et al., 2015) and the functions it covered were diverse such as multiple signaling pathways and signaling molecules were activated; reconfiguration of metabolism was started; and the developmental process was repressed. Several studies have evidenced that OSB could modulate certain defense responses of plants (Chung et al., 2013; Acevedo et al., 2017), and significantly, our study broadened the understanding of how OSB modulated potato defense response from a macroscopic perspective. OSB-derived HACs broadly modulated potato defense response; some early signaling were activated; photosynthesis and its related process were repressed; phenylpropanoid biosynthesis was inhibited, etc. The identification of these gene modules will help determine which herbivory components regulate to-be-studied herbivory-responding genes.

While we revealed how potato plants integrated the responses to herbivory components *via* accumulatively adding the components, the impact of individual and mixed components on potato defense response could be different. Thus, the impact of OSB-derived HACs alone on potato defense response remains to be characterized in future. Moreover, many defense hormones and genes associated with early response to insect herbivory were strongly upregulated within a few hours after simulated herbivory in solanaceous plants (Durrant et al., 2017). We also found that the key defense phytohormones JA and JA-Ile were significantly upregulated at 1 h after elicitation in potato plants (Supplementary Figure 6). It would be interesting to study the transcriptomic response in a kinetic way with more time points in future to get a broad picture for potato early response. In addition, our study disentangled PTM herbivory at the level of functional components; however, each functional component includes multiple cues playing different role in response induction (Schmelz et al., 2009). The future studies should focus on identifying key cues in these components such as specific elicitor/suppresser of bacteria or specific chemical cues in oral secretion and clarifying the mechanism of how these key cues induce or modulate potato defense response.

## Data Availability Statement

The datasets presented in this study can be found in online repositories. The names of the repository/repositories and accession number(s) can be found in the article/Supplementary Material.

## Author Contributions

WZ and ZM contributed to conception and design of the study. ZM and YG carried out the experiment. ZM performed the statistical analysis. YZ, JZ, and AM wrote sections of the manuscript. WZ and ZZ contributed to manuscript revision, read, and approved the submitted version. All authors contributed to the article and approved the submitted version.

## Conflict of Interest

The authors declare that the research was conducted in the absence of any commercial or financial relationships that could be construed as a potential conflict of interest.

## Publisher’s Note

All claims expressed in this article are solely those of the authors and do not necessarily represent those of their affiliated organizations, or those of the publisher, the editors and the reviewers. Any product that may be evaluated in this article, or claim that may be made by its manufacturer, is not guaranteed or endorsed by the publisher.

## Supplementary Material

Supplementary Figure 1 Antibiotic cocktail largely reduced bacterial colonies in PTM oral secretion. OS_PTM_ was treated with antibiotic cocktail as the following protocol: **(A)** 200 μl cocktail was evenly wiped on detached mature potato leaf with sterile gloves, **(B)** the leaf was placed in chemical hood until it dries, **(C)** third-instar PTM larvae (starved for 12 h) was fed with AB-treated potato leaf for 48 h and potato leaf was replaced every 24 h, **(D)** the AB-OS_PTM_ was collected after 48-h feeding. OS_PTM_ was collected the same as AB-OS_PTM_ but potato leaf was treated with Milli-Q water. **(A)** OS_PTM_ and AB-OS_PTM_ were diluted 1:1,000,000 v/v with Milli-Q water and 100 μl diluent was cultured in 2× YT agar plate overnight. **(B)** Colony numbers of two plates are exhibited as a bar plot.

Supplementary Figure 2 The inductive similarity between actual herbivory and simulated herbivory in defense-related GOs. The DEG number of simulated herbivory and actual herbivory in GOs of **(A)** photosynthesis, **(B)** primary metabolism, **(C)** secondary metabolism, **(D)** molecular function, and **(E)** cellular component are exhibited as bar plots; the similarity is mapped to heatmap color from gray to red.

Supplementary Figure 3 The *Z*-score standardized expression of key genes of **(A)** JA and **(B)** ABA pathway.

Supplementary Figure 4 Functional network of molecular function GOs for seven gene modules. **(A)** Top 15 overrepresented molecular function GOs of each gene module were integrated as a functional network. Node refers to the GO term, and the edge refers to that two GOs were functionally connected; *p*-value of overrepresentation analysis is mapped to node size; Jaccard similarity between two GO terms is mapped to edge width; gene module is mapped to node color. **(B)** Gene modules were clustered based on topological overlap; it cut gene modules into four clusters. **(C)** The intramodular connectivity and extramodular connectivity of gene modules are displayed as a dot plot.

Supplementary Figure 5 The verification of RNA-seq with quantitative real-time PCR (qRT-PCR). Four key genes (*StCOI1*, *StGAL83*, *StPAL1*, and *StEIN3*) that involved in potato response to insect herbivory were selected for qRT-PCR verification; these genes were upregulated or downregulated under insect herbivory. Blue bar plots (mean ± SE) represent the gene expression in RNA-seq and gray bar plots (mean ± SE) represent the gene expression in qRT-PCR.

Supplementary Figure 6 Temporal dynamic of phytohormones in potato plants after simulated herbivory. Four phytohormones JA **(A)**, JA-Ile **(B)**, ABA **(C)**, and SA **(D)** were measured at 0, 0.5, 1, and 3 h (mean ± SE, *n* = 5). Asterisks indicate significant differences between treatment and control (Student’s *t*-test, **p* < 0.05, ***p* < 0.01, and ****p* < 0.001).

Supplementary Figure 7 Phytohormonal responses were not affected by antibiotic cocktail at 1 h after elicitation. Four phytohormone JA **(A)**, JA-Ile **(B)**, ABA **(C)**, and SA **(D)** were measured at 1 h after W + W (wounding + water), W + AB diluent (wounding + antibiotic cocktail 1:5 diluent), and W + AB (wounding + antibiotic cocktail). One-way ANOVA results for each phytohormone are shown (ns, not significant).

Supplementary Table 1 The primers for qRT-PCR in this study.

Supplementary Datasheet 1 Gene information of each gene module.

Supplementary Datasheet 2 Read count matrix of RNA-seq and GO enrichment analysis result for each gene module.

## References

[B1] Acevedo F. E. Peiffer M. Tan C. W. Stanley B. A. Stanley A. Wang J. (2017). Fall armyworm-associated gut bacteria modulate plant defense responses. *Mol. Plant Microbe Interact.* 30 127–137. 10.1094/MPMI-11-16-0240-R 28027025

[B2] Acevedo F. E. Rivera-Vega L. J. Chung S. H. Ray S. Felton G. W. (2015). Cues from chewing insects – the intersection of DAMPs, HAMPs, MAMPs and effectors. *Curr. Opin. Plant Biol.* 26 80–86. 10.1016/j.pbi.2015.05.029 26123394

[B3] Alborn H. T. Hansen T. V. Jones T. H. Bennett D. C. Tumlinson J. H. Schmelz E. A. (2007). Disulfooxy fatty acids from the American bird grasshopper *Schistocerca americana*, elicitors of plant volatiles. *Proc. Natl. Acad. Sci. U.S.A.* 104 12976–12981. 10.1073/pnas.0705947104 17664416 PMC1941812

[B4] Alborn H. T. Turlings T. C. J. Jones T. H. Stenhagen G. Loughrin J. H. Tumlinson J. H. (1997). An elicitor of plant volatiles from beet armyworm oral secretion. *Science* 276 945–949. 10.1126/science.276.5314.945

[B5] Altmann M. Altmann S. Rodriguez P. A. Weller B. Elorduy Vergara L. Palme J. (2020). Extensive signal integration by the phytohormone protein network. *Nature* 583 271–276. 10.1038/s41586-020-2460-0 32612234

[B6] Appel H. M. Cocroft R. B. (2014). Plants respond to leaf vibrations caused by insect herbivore chewing. *Oecologia* 175 1257–1266. 10.1007/s00442-014-2995-6 24985883 PMC4102826

[B7] Bjornson M. Pimprikar P. Nurnberger T. Zipfel C. (2021). The transcriptional landscape of Arabidopsis thaliana pattern-triggered immunity. *Nat. Plants* 7 579–586. 10.1038/s41477-021-00874-5 33723429 PMC7610817

[B8] Bodenhausen N. Reymond P. (2007). Signaling pathways controlling induced resistance to insect herbivores in *Arabidopsis*. *Mol. Plant Microbe Interact.* 20 1406–1420. 10.1094/Mpmi-20-11-1406 17977152

[B9] Chandel R. S. Vashisth S. Soni S. Kumar R. Kumar V. (2020). The potato tuber moth, *Phthorimaea operculella* (Zeller), in India: biology, ecology, and control. *Potato Res.* 63 15–39. 10.1007/s11540-019-09426-z

[B10] Choi J. Tanaka K. Cao Y. R. Qi Y. Qiu J. Liang Y. (2014). Identification of a plant receptor for extracellular ATP. *Science* 343 290–294. 10.1126/science.343.6168.290 24436418

[B11] Christensen S. A. Nemchenko A. Borrego E. Murray I. Sobhy I. S. Bosak L. (2013). The maize lipoxygenase, ZmLOX10, mediates green leaf volatile, jasmonate and herbivore-induced plant volatile production for defense against insect attack. *Plant J.* 74 59–73. 10.1111/tpj.12101 23279660

[B12] Chung S. H. Rosa C. Scully E. D. Peiffer M. Tooker J. F. Hoover K. (2013). Herbivore exploits orally secreted bacteria to suppress plant defenses. *Proc. Natl. Acad. Sci. U.S.A.* 110 15728–15733. 10.1073/pnas.1308867110 24019469 PMC3785742

[B13] Durrant M. Boyer J. Zhou W. W. Baldwin I. T. Xu S. Q. (2017). Evidence of an evolutionary hourglass pattern in herbivory-induced transcriptomic responses. *New Phytol.* 215 1264–1273. 10.1111/nph.14644 28618009

[B14] Erb M. Meldau S. Howe G. A. (2012). Role of phytohormones in insect-specific plant reactions. *Trends Plant Sci.* 17 250–259. 10.1016/j.tplants.2012.01.003 22305233 PMC3346861

[B15] Erb M. Reymond P. (2019). Molecular interactions between plants and insect herbivores. *Annu. Rev. Plant. Biol.* 70 527–557. 10.1146/annurev-arplant-050718-095910 30786233

[B16] Farmer E. E. Dubugnon L. (2009). Detritivorous crustaceans become herbivores on jasmonate-deficient plants. *Proc. Natl. Acad. Sci. U.S.A.* 106 935–940. 10.1073/pnas.0812182106 19139394 PMC2630106

[B17] Gentleman R. C. Carey V. J. Bates D. M. Bolstad B. Dettling M. Dudoit S. (2004). Bioconductor: open software development for computational biology and bioinformatics. *Genome Biol.* 5:R80. 10.1186/gb-2004-5-10-r80 15461798 PMC545600

[B18] Halitschke R. Schittko U. Pohnert G. Boland W. Baldwin I. T. (2001). Molecular interactions between the specialist herbivore *Manduca sexta* (Lepidoptera, Sphingidae) and its natural host *Nicotiana attenuata*. III. Fatty acid-amino acid conjugates in herbivore oral secretions are necessary and sufficient for herbivore-specific plant responses. *Plant Physiol.* 125 711–717. 10.1104/pp.125.2.711 11161028 PMC64872

[B19] Horgan F. G. Quiring D. T. Lagnaoui A. Pelletier Y. (2007). Variable responses of tuber moth to the leaf trichomes of wild potatoes. *Entomol. Exp. Appl.* 125 1–12. 10.1111/j.1570-7458.2007.00590.x

[B20] Kang J. H. Wang L. Giri A. Baldwin I. T. (2006). Silencing threonine deaminase and JAR4 in *Nicotiana attenuata* impairs jasmonic acid-isoleucine-mediated defenses against *Manduca sexta*. *Plant Cell* 18 3303–3320. 10.1105/tpc.106.041103 17085687 PMC1693959

[B21] Kazan K. Lyons R. (2014). Intervention of phytohormone pathways by pathogen effectors. *Plant Cell* 26 2285–2309. 10.1105/tpc.114.125419 24920334 PMC4114936

[B22] Kollasch A. M. Abdul-Kafi A. R. Body M. J. A. Pinto C. F. Appel H. M. Cocroft R. B. (2020). Leaf vibrations produced by chewing provide a consistent acoustic target for plant recognition of herbivores. *Oecologia* 194 1–13. 10.1007/s00442-020-04672-2 32533358

[B23] Koo A. J. K. Howe G. A. (2009). The wound hormone jasmonate. *Phytochemistry* 70 1571–1580. 10.1016/j.phytochem.2009.07.018 19695649 PMC2784233

[B24] Lin P. A. Chen Y. T. Chaverra-Rodriguez D. Heu C. C. Bin Zainuddin N. Sidhu J. S. (2021). Silencing the alarm: an insect salivary enzyme closes plant stomata and inhibits volatile release. *New Phytol.* 230 793–803. 10.1111/nph.17214 33459359 PMC8048682

[B25] Love M. I. Huber W. Anders S. (2014). Moderated estimation of fold change and dispersion for RNA-seq data with DESeq2. *Genome Biol.* 15:550. 10.1186/s13059-014-0550-8 25516281 PMC4302049

[B26] Luo J. Wei K. Wang S. H. Zhao W. Y. Ma C. R. Hettenhausen C. (2016). COI1-regulated hydroxylation of jasmonoyl-L-isoleucine impairs *Nicotiana attenuata’s* resistance to the generalist herbivore *Spodoptera litura*. *J. Agric. Food Chem.* 64 2822–2831. 10.1021/acs.jafc.5b06056 26985773

[B27] Merz D. Richter J. Gonneau M. Sanchez-Rodriguez C. Eder T. Sormani R. (2017). T-DNA alleles of the receptor kinase THESEUS1 with opposing effects on cell wall integrity signaling. *J. Exp. Bot.* 68 4583–4593. 10.1093/jxb/erx263 28981771 PMC5853656

[B28] Musmeci S. Ciccoli R. Di Gioia V. Sonnino A. Arnone S. (1997). Leaf effects of wild species of *Solanum* and interspecific hybrids on growth and behaviour of the potato tuber moth, *Phthorimaea operculella* Zeller. *Potato Res.* 40 417–430. 10.1007/Bf02358002

[B29] Mweetwa A. M. Hunter D. Poe R. Harich K. C. Ginzberg I. Veilleux R. E. (2012). Steroidal glycoalkaloids in *Solanum chacoense*. *Phytochemistry* 75 32–40. 10.1016/j.phytochem.2011.12.003 22217745

[B30] Petersen M. Brodersen P. Naested H. Andreasson E. Lindhart U. Johansen B. (2000). *Arabidopsis* MAP kinase 4 negatively regulates systemic acquired resistance. *Cell* 103 1111–1120. 10.1016/S0092-8674(00)00213-0 11163186

[B31] Pieterse C. M. Van der Does D. Zamioudis C. Leon-Reyes A. Van Wees S. C. (2012). Hormonal modulation of plant immunity. *Annu. Rev. Cell Dev. Biol.* 28 489–521. 10.1146/annurev-cellbio-092910-154055 22559264

[B32] Rani M. Kumar S. Shukla S. Sachan M. Sharkar B. (2012). Comparative study of effect of different tissue culture media on the propagation of potato. *Res. Rev. J. Microbiol. Biotechnol.* 1 12–14.

[B33] Robert-Seilaniantz A. Grant M. Jones J. D. G. (2011). Hormone crosstalk in plant disease and defense: more than just jasmonate-salicylate antagonism. *Annu. Rev. Phytopathol.* 49 317–343. 21663438 10.1146/annurev-phyto-073009-114447

[B34] Schmelz E. A. Carroll M. J. LeClere S. Phipps S. M. Meredith J. Chourey P. S. (2006). Fragments of ATP synthase mediate plant perception of insect attack. *Proc. Natl. Acad. Sci. U.S.A.* 103 8894–8899. 10.1073/pnas.0602328103 16720701 PMC1482674

[B35] Schmelz E. A. Engelberth J. Alborn H. T. Tumlinson J. H. Teal P. E. A. (2009). Phytohormone-based activity mapping of insect herbivore-produced elicitors. *Proc. Natl. Acad. Sci. U.S.A.* 106 653–657. 10.1073/pnas.0811861106 19124770 PMC2626758

[B36] Schmelz E. A. LeClere S. Carroll M. J. Alborn H. T. Teal P. E. A. (2007). Cowpea chloroplastic ATP synthase is the source of multiple plant defense elicitors during insect herbivory. *Plant Physiol.* 144 793–805. 10.1104/pp.107.097154 17369425 PMC1914193

[B37] Schuman M. C. Baldwin I. T. (2016). The layers of plant responses to insect herbivores. *Annu. Rev. Entomol.* 61 373–394. 10.1146/annurev-ento-010715-023851 26651543

[B38] Schweizer F. Fernandez-Calvo P. Zander M. Diez-Diaz M. Fonseca S. Glauser G. (2013). Arabidopsis basic helix-loop-helix transcription factors MYC2, MYC3, and MYC4 regulate glucosinolate biosynthesis, insect performance, and feeding behavior. *Plant Cell* 25 3117–3132. 10.1105/tpc.113.115139 23943862 PMC3784603

[B39] Song S. S. Huang H. Gao H. Wang J. J. Wu D. W. Liu X. L. (2014). Interaction between MYC2 and ETHYLENE INSENSITIVE3 modulates antagonism between jasmonate and ethylene signaling in *Arabidopsis*. *Plant Cell* 26 263–279. 10.1105/tpc.113.120394 24399301 PMC3963574

[B40] Spoel S. H. Koornneef A. Claessens S. M. C. Korzelius J. P. Van Pelt J. A. Mueller M. J. (2003). NPR1 modulates cross-talk between salicylate- and jasmonate-dependent defense pathways through a novel function in the cytosol. *Plant Cell* 15 760–770. 10.1105/tpc.009159 12615947 PMC150028

[B41] Thaler J. S. Bostock R. M. (2004). Interactions between abscisic-acid-mediated responses and plant resistance to pathogens and insects. *Ecology* 85 48–58. 10.1890/02-0710

[B42] Thaler J. S. Humphrey P. T. Whiteman N. K. (2012). Evolution of jasmonate and salicylate signal crosstalk. *Trends Plant Sci.* 17 260–270. 10.1016/j.tplants.2012.02.010 22498450

[B43] The Potato Genome Sequencing Consortium (2011). Genome sequence and analysis of the tuber crop potato. *Nature* 475 189–195. 10.1038/nature10158 21743474

[B44] Toruno T. Y. Stergiopoulos I. Coaker G. (2016). Plant-pathogen effectors: cellular probes interfering with plant defenses in spatial and temporal manners. *Annu. Rev. Phytopathol.* 54 419–441. 10.1146/annurev-phyto-080615-100204 27359369 PMC5283857

[B45] Valladares F. Sanchez-Gomez D. Zavala M. A. (2006). Quantitative estimation of phenotypic plasticity: bridging the gap between the evolutionary concept and its ecological applications. *J. Ecol.* 94 1103–1116. 10.1111/j.1365-2745.2006.01176.x

[B46] Verma V. Ravindran P. Kumar P. P. (2016). Plant hormone-mediated regulation of stress responses. *BMC Plant Biol.* 16:86. 10.1186/s12870-016-0771-y 27079791 PMC4831116

[B47] Vogt T. (2010). Phenylpropanoid biosynthesis. *Mol. Plant* 3 2–20. 10.1093/mp/ssp106 20035037

[B48] Wang J. Peiffer M. Hoover K. Rosa C. Zeng R. Felton G. W. (2017). *Helicoverpa zea* gut-associated bacteria indirectly induce defenses in tomato by triggering a salivary elicitor(s). *New Phytol.* 214 1294–1306. 10.1111/nph.14429 28170113

[B49] Wang L. Halitschke R. Kang J.-H. Berg A. Harnisch F. Baldwin I. T. (2007). Independently silencing two JAR family members impairs levels of trypsin proteinase inhibitors but not nicotine. *Planta* 226 159–167. 10.1007/s00425-007-0477-3 17273867

[B50] Waterman J. M. Cazzonelli C. I. Hartley S. E. Johnson S. N. (2019). Simulated herbivory: the key to disentangling plant defence responses. *Trends Ecol. Evol.* 34 447–458. 10.1016/j.tree.2019.01.008 30824196

[B51] Yu G. Wang L. G. Han Y. He Q. Y. (2012). clusterProfiler: an R package for comparing biological themes among gene clusters. *OMICS* 16 284–287. 10.1089/omi.2011.0118 22455463 PMC3339379

[B52] Zhang B. Horvath S. (2005). A general framework for weighted gene co-expression network analysis. *Stat. Appl. Genet. Mol. Biol.* 4:Article17. 10.2202/1544-6115.1128 16646834

[B53] Zhang M. D. Yan J. J. Ali A. Gao Y. L. (2021). Potato plant variety affects the performance and oviposition preference of *Phthorimaea operculella* Zeller (Lepidoptera: Gelechiidae). *Pest Manag. Sci.* 10.1002/ps.6625 [Epub ahead of print]. 34477288

[B54] Zhou W. W. Brochmoller T. Ling Z. H. Omdahl A. Baldwin I. T. Xu S. Q. (2016). Evolution of herbivore-induced early defense signaling was shaped by genome wide duplications in *Nicotiana*. *Elife* 5:e19531. 10.7554/eLife.19531 27813478 PMC5115867

