# Supplementary figures and images for: Disentangling the Potato Tuber Moth-Induced Early-Defense Response by Simulated Herbivory in Potato Plants
Source: Front Plant Sci. 2022 May 26;13:902342. doi: 10.3389/fpls.2022.902342 (PMC9178332; doi:10.3389/fpls.2022.902342)

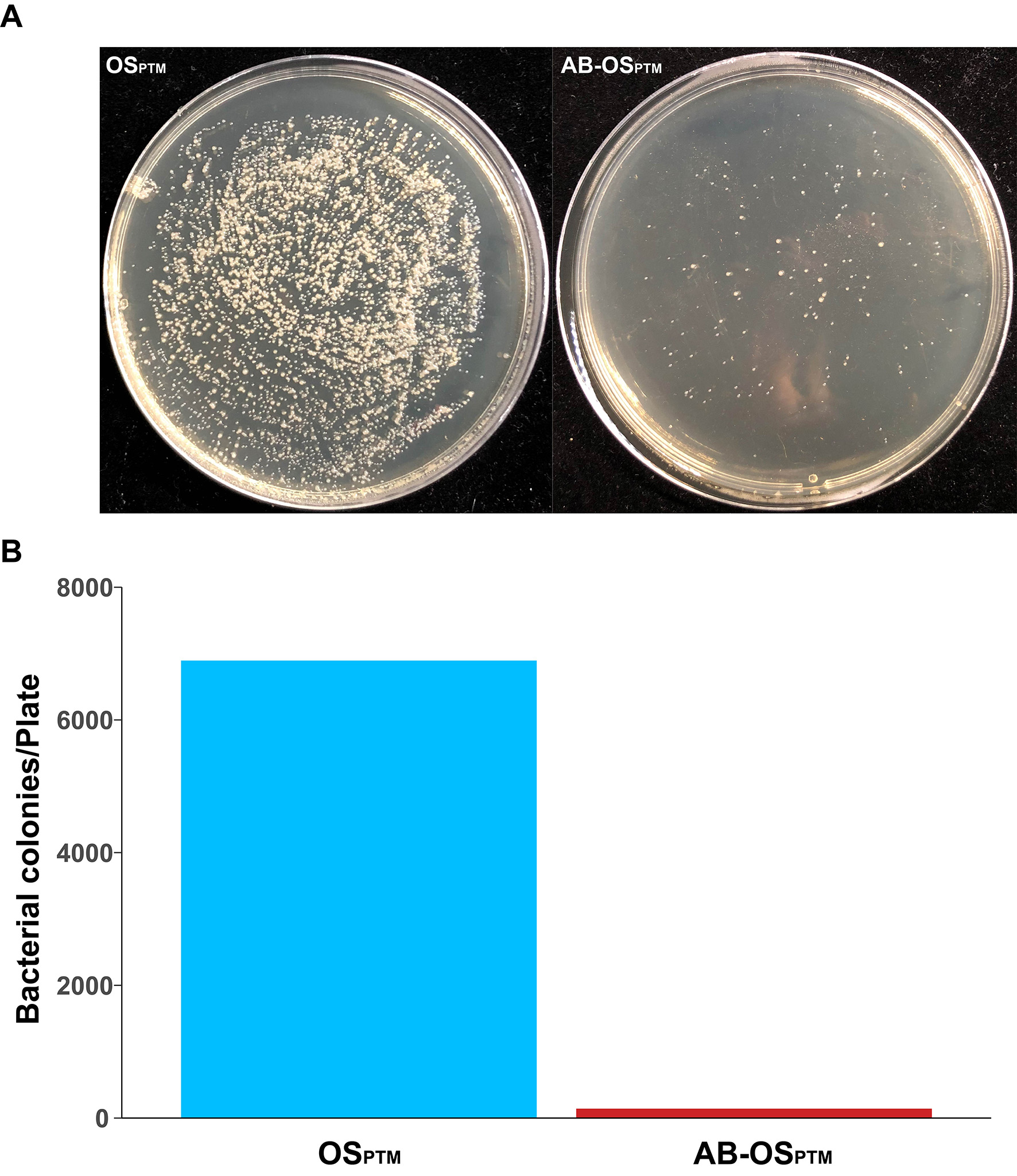

Supplement: Supplementary Figure 1 — Antibiotic cocktail largely reduced bacterial colonies in PTM oral secretion. OSPTM was treated with antibiotic cocktail as the following protocol: (A) 200 μl cocktail was evenly wiped on detached mature potato leaf with sterile gloves, (B) the leaf was placed in chemical hood until it dries, (C) third-instar PTM larvae (starved for 12 h) was fed with AB-treated potato leaf for 48 h and potato leaf was replaced every 24 h, (D) the AB-OSPTM was collected after 48-h feeding. OSPTM was collected the same as AB-OSPTM but potato leaf was treated with Milli-Q water. (A) OSPTM and AB-OSPTM were diluted 1:1,000,000 v/v with Milli-Q water and 100 μl diluent was cultured in 2× YT agar plate overnight. (B) Colony numbers of two plates are exhibited as a bar plot. [file Image_1.JPEG]

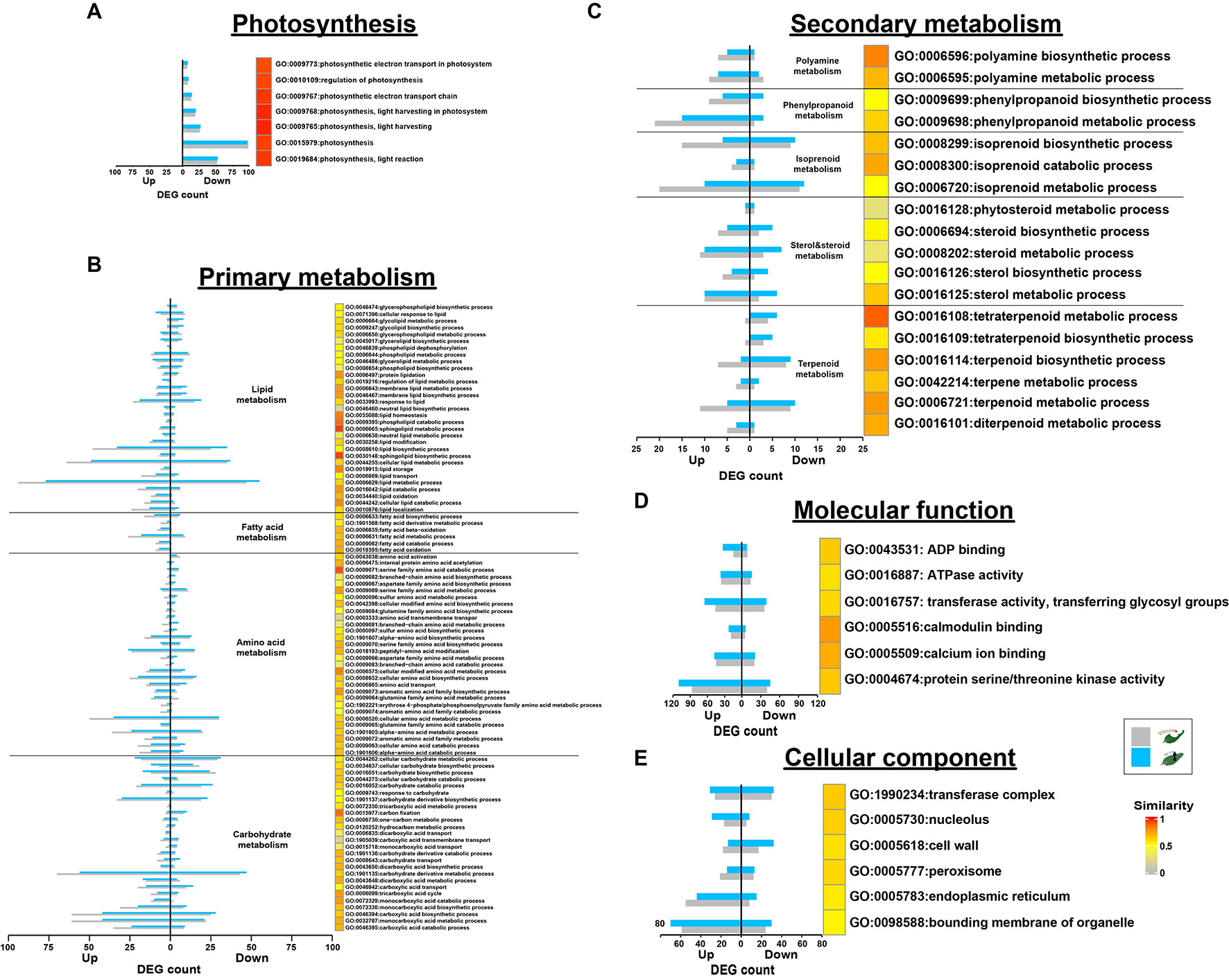

Supplement: Supplementary Figure 2 — The inductive similarity between actual herbivory and simulated herbivory in defense-related GOs. The DEG number of simulated herbivory and actual herbivory in GOs of (A) photosynthesis, (B) primary metabolism, (C) secondary metabolism, (D) molecular function, and (E) cellular component are exhibited as bar plots; the similarity is mapped to heatmap color from gray to red. [file Image_2.JPEG]

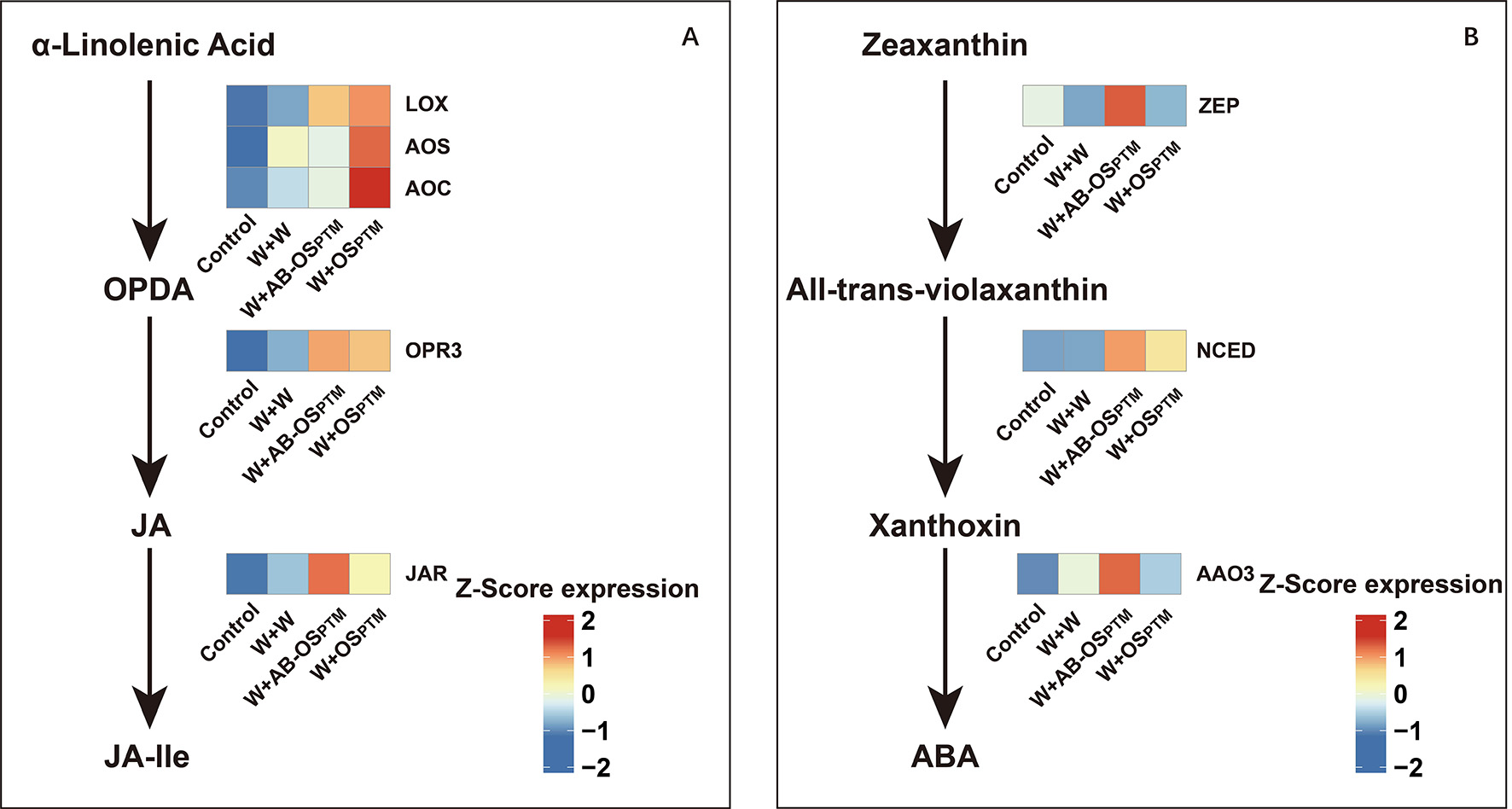

Supplement: Supplementary Figure 3 — The Z-score standardized expression of key genes of (A) JA and (B) ABA pathway. [file Image_3.JPEG]

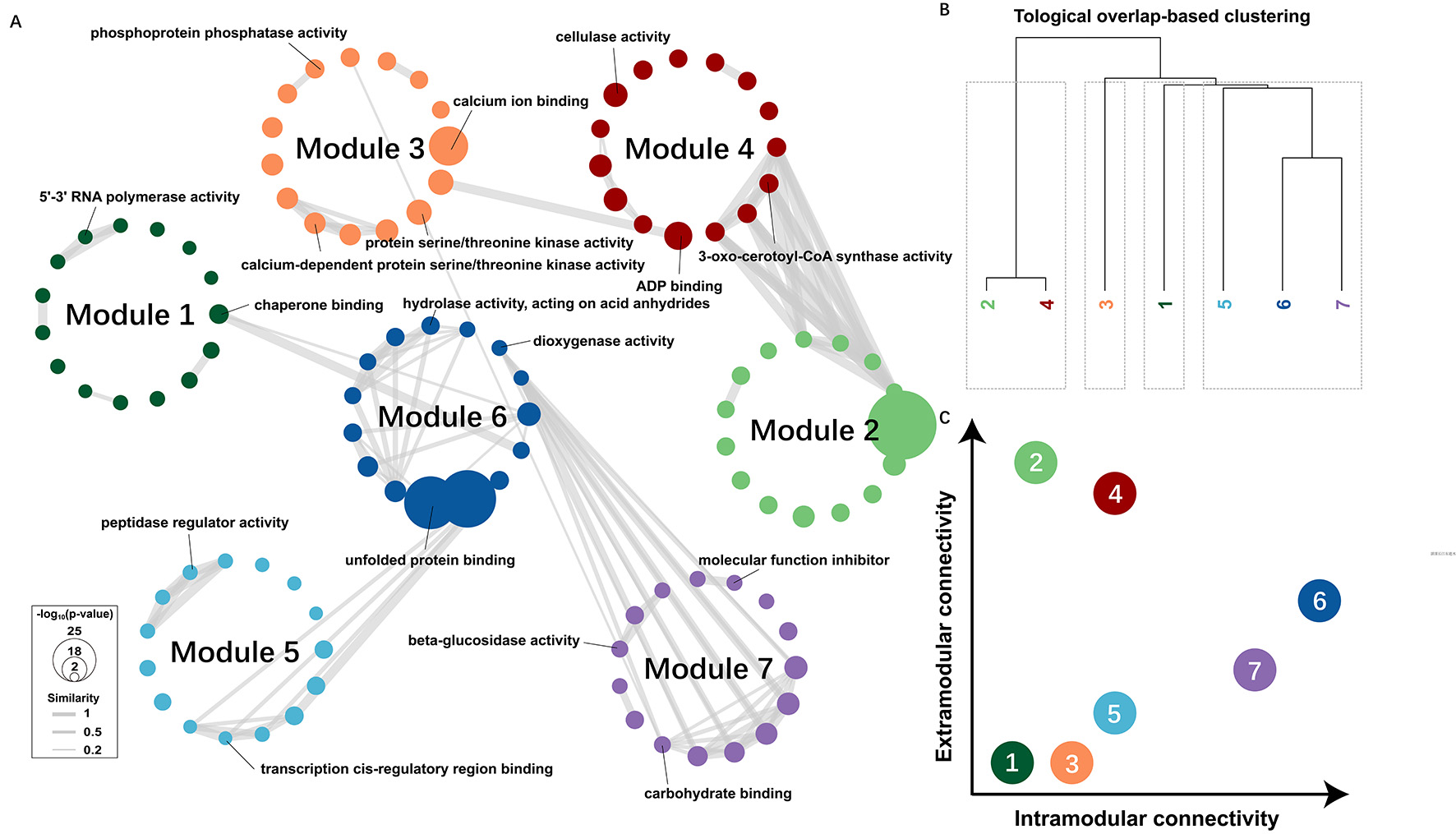

Supplement: Supplementary Figure 4 — Functional network of molecular function GOs for seven gene modules. (A) Top 15 overrepresented molecular function GOs of each gene module were integrated as a functional network. Node refers to the GO term, and the edge refers to that two GOs were functionally connected; p-value of overrepresentation analysis is mapped to node size; Jaccard similarity between two GO terms is mapped to edge width; gene module is mapped to node color. (B) Gene modules were clustered based on topological overlap; it cut gene modules into four clusters. (C) The intramodular connectivity and extramodular connectivity of gene modules are displayed as a dot plot. [file Image_4.JPEG]

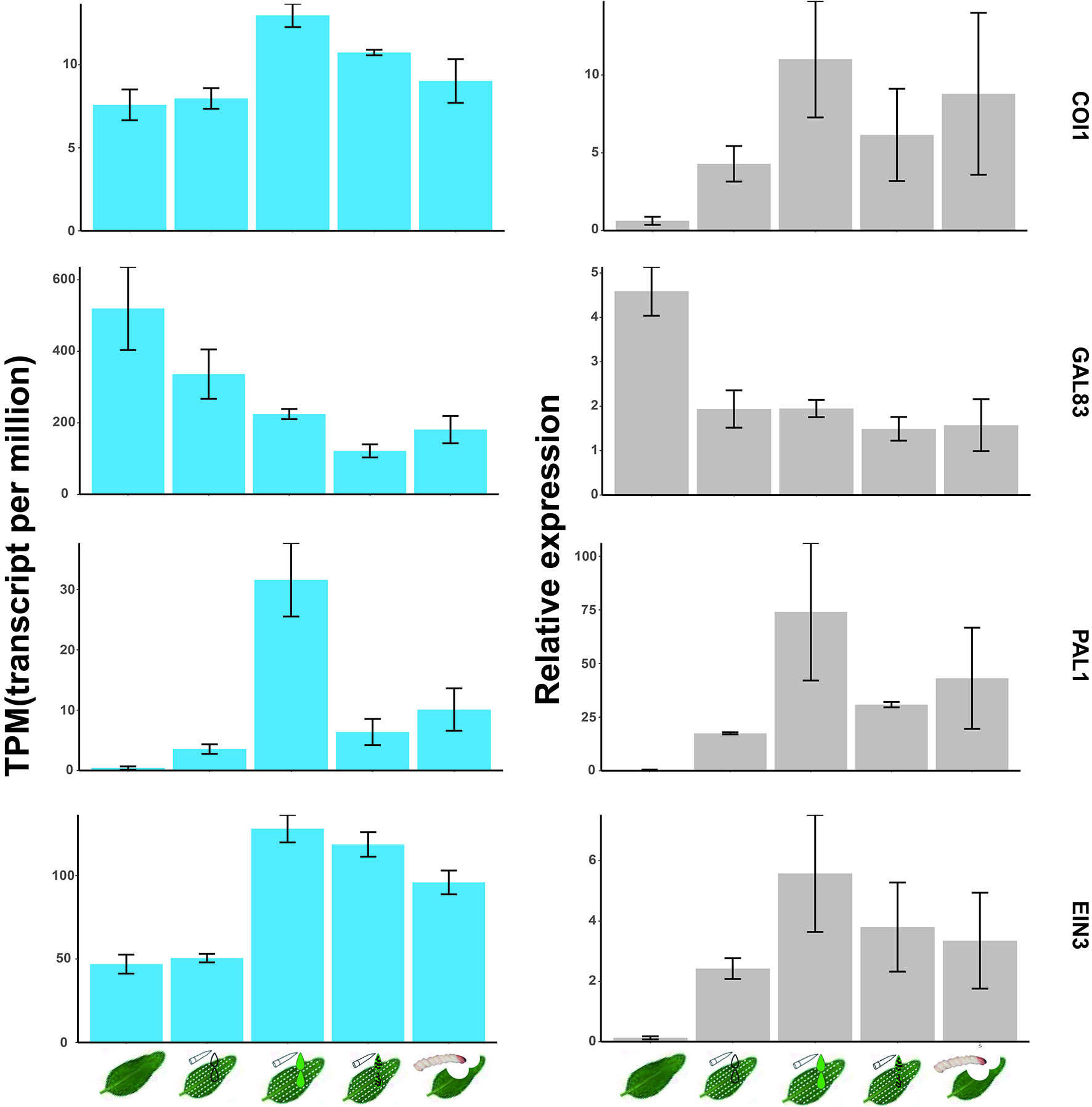

Supplement: Supplementary Figure 5 — The verification of RNA-seq with quantitative real-time PCR (qRT-PCR). Four key genes (StCOI1, StGAL83, StPAL1, and StEIN3) that involved in potato response to insect herbivory were selected for qRT-PCR verification; these genes were upregulated or downregulated under insect herbivory. Blue bar plots (mean ± SE) represent the gene expression in RNA-seq and gray bar plots (mean ± SE) represent the gene expression in qRT-PCR. [file Image_5.JPEG]

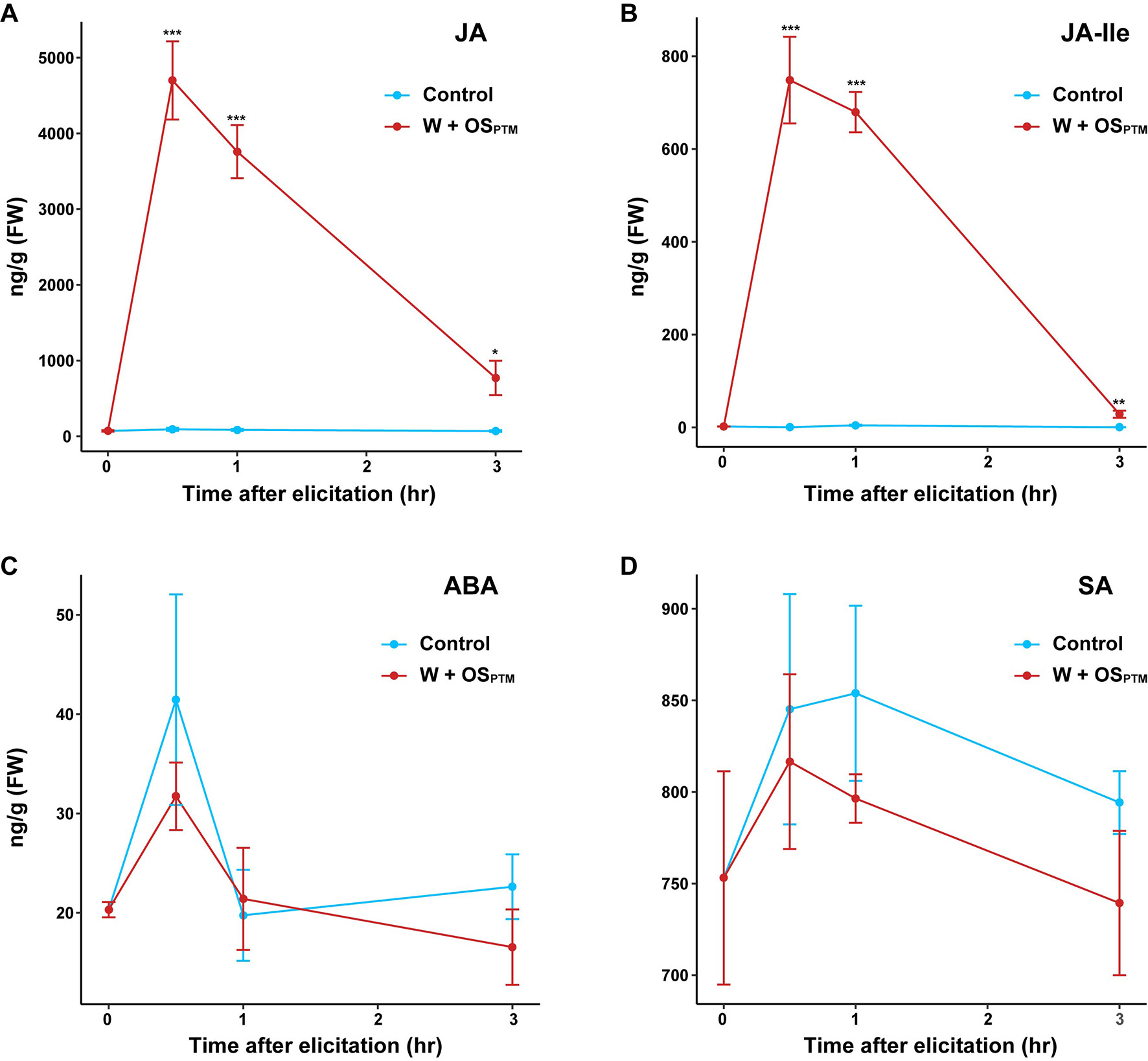

Supplement: Supplementary Figure 6 — Temporal dynamic of phytohormones in potato plants after simulated herbivory. Four phytohormones JA (A), JA-Ile (B), ABA (C), and SA (D) were measured at 0, 0.5, 1, and 3 h (mean ± SE, n = 5). Asterisks indicate significant differences between treatment and control (Student’s t-test, *p < 0.05, **p < 0.01, and ***p < 0.001). [file Image_6.JPEG]

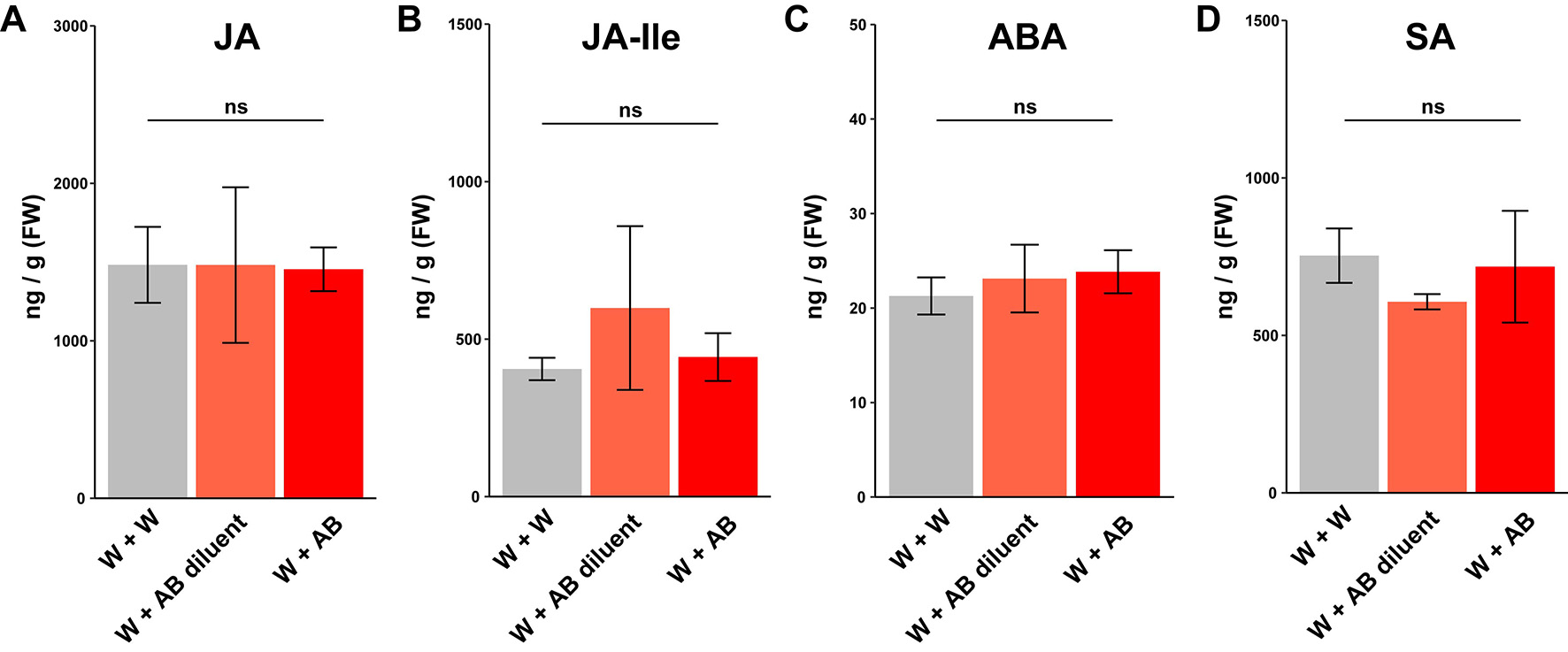

Supplement: Supplementary Figure 7 — Phytohormonal responses were not affected by antibiotic cocktail at 1 h after elicitation. Four phytohormone JA (A), JA-Ile (B), ABA (C), and SA (D) were measured at 1 h after W + W (wounding + water), W + AB diluent (wounding + antibiotic cocktail 1:5 diluent), and W + AB (wounding + antibiotic cocktail). One-way ANOVA results for each phytohormone are shown (ns, not significant). [file Image_7.JPEG]
