# Supplementary material for: Disentangling the Potato Tuber Moth-Induced Early-Defense Response by Simulated Herbivory in Potato Plants
Source: Front Plant Sci. 2022 May 26;13:902342. doi: 10.3389/fpls.2022.902342 (PMC9178332; doi:10.3389/fpls.2022.902342)
Supplement: Supplementary Table 1 — The primers for qRT-PCR in this study. [file Table_1.DOCX]

| **Primer** | **Sequence** |
| --- | --- |
| EF3d-F | GGAGCACAGGAGAAGATGAAGGAG |
| EF3d-R | CGTTGGTGAATGCGGCAGTAGG |
| COI1-F | TGCCGCACTAGTTTTCCCTC |
| COI1-R | GCTGCTGTGTCAAGAAGTGC |
| EIN3-F | GGGGAATGTTGCAGTGACCA |
| EIN3-R | CAGGATTGCTTTGTGGGTGC |
| PAL-F | TGGTCAGATTGAGGCTGCTG |
| PAL-R | AGGGGTTCCTTGGAAGTTGC |
| GAL83-F | AGATCAGGCAAGGACTATACCGT |
| GAL83-R | CGAAATCCTCATCTCCCAGCAA |
